# Bis[2-(1*H*-benzimidazol-2-yl)phenolato-κ^2^ *N* ^3^,*O*]cobalt(II) dimethyl­formamide disolvate

**DOI:** 10.1107/S1600536808029620

**Published:** 2008-09-21

**Authors:** Feigang He, Yun Xi, Jun Li, Fengxing Zhang

**Affiliations:** a Department of Chemistry, Shannxi Institute of Education, Xi’an, Shaanxi 710061, People’s Republic of China; b Department of Chemistry, Northwest University, Xi’an, Shaanxi 710069, People’s Republic of China

## Abstract

In the crystal structure of the title compound, [Co(C_13_H_9_N_2_O)_2_]·2C_3_H_7_NO, the Co^II^ ion is four-coordinated by two N atoms and two O atoms from two deprotonated 2-(1*H*-benzimidazol-2-yl)phenol ligands in a distorted tetra­hedral geometry. The dimethyl­formamide solvent mol­ecules are found inside a two-dimensional network structure formed by inter­molecular N—H⋯O hydrogen bonds linking the mol­ecules.

## Related literature

For related literature, see: Benzekri *et al.* (1991 ▶); Crane *et al.* (1995 ▶); Lorosch & Haase (1985 ▶); Maekawa *et al.* (1989 ▶); McKee *et al.* (1981 ▶); Sundburg & Martin (1974 ▶); Nalwa *et al.* (2003 ▶) and references cited therein; Tong *et al.* (2005 ▶).
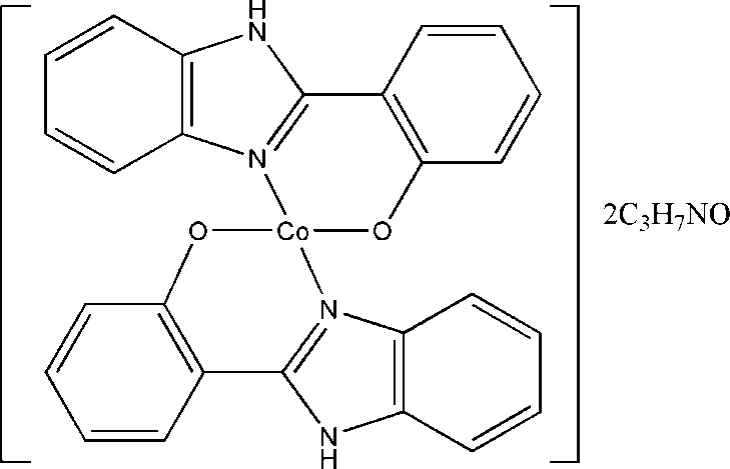


## Experimental

### 

#### Crystal data

[Co(C_13_H_9_N_2_O)_2_]·2C_3_H_7_NO
*M* *_r_* = 623.57
Orthorhombic, 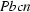

*a* = 15.440 (2) Å
*b* = 8.7022 (12) Å
*c* = 22.156 (3) Å
*V* = 2977.0 (7) Å^3^
*Z* = 4
Mo *K*α radiation
μ = 0.62 mm^−1^
*T* = 298 (2) K
0.34 × 0.28 × 0.09 mm

#### Data collection

Bruker SMART CCD area-detector diffractometer
Absorption correction: multi-scan (*ABSCOR*; Higashi, 1995 ▶) *T*_min_ = 0.816, *T*_max_ = 0.946
12837 measured reflections
3157 independent reflections
2167 reflections with *I* > 2σ(*I*)
*R*_int_ = 0.036

#### Refinement

*R*[*F* ^2^ > 2σ(*F* ^2^)] = 0.038
*wR*(*F* ^2^) = 0.107
*S* = 1.03
3157 reflections
197 parameters
H-atom parameters constrained
Δρ_max_ = 0.24 e Å^−3^
Δρ_min_ = −0.34 e Å^−3^

### 

Data collection: *SMART* (Bruker, 2000 ▶); cell refinement: *SAINT* (Bruker, 2000 ▶); data reduction: *SAINT*; program(s) used to solve structure: *SHELXS97* (Sheldrick, 2008 ▶); program(s) used to refine structure: *SHELXL97* (Sheldrick, 2008 ▶); molecular graphics: *SHELXTL* (Sheldrick, 2008 ▶); software used to prepare material for publication: *SHELXTL*.

## Supplementary Material

Crystal structure: contains datablocks I, global. DOI: 10.1107/S1600536808029620/bv2101sup1.cif

Structure factors: contains datablocks I. DOI: 10.1107/S1600536808029620/bv2101Isup2.hkl

Additional supplementary materials:  crystallographic information; 3D view; checkCIF report

## Figures and Tables

**Table 1:** Hydrogen-bond geometry (Å, °)

*D*—H⋯*A*	*D*—H	H⋯*A*	*D*⋯*A*	*D*—H⋯*A*
N2—H2*A*⋯O2	0.86	1.94	2.772 (2)	164

**Fig. 1.**
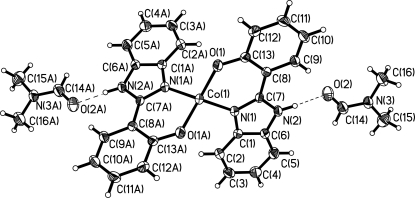
A view of the molecular structure of (I) with the atom-numbering scheme. Displacement ellipsoids are drawn at the 50% probability level for non-hydrogen atoms. H atoms are shown as small spheres of arbitrary radii. [symmetry code: (i) -x + 1, y, -z + 1/2]

**Fig. 2.**
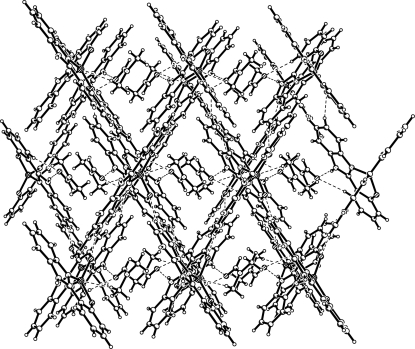
Two-dimensional network structure with the solvent molecules encapsulated.

## supplementary crystallographic information

### Comment

Complexes with imidazole-related and imidazole-containing ligands serve as models for metalloproteins and have been extensively studied in recent years (Sundburg *et al.*, 1974; Maekawa *et al.*, 1989; Lorosch & Haase, 1985; Benzekri *et al.*, 1991; Crane *et al.*, 1995; McKee *et al.*, 1981). It has been shown in the previous reports, that the benzimidazole-based derivatives are a novel class of N,*O*-donor ligands that could form sublimable luminescent complexes as possible electroluminescent materials (Nalwa *et al.*, 2003; Tong *et al.*, 2005). The bidentate ligand 2-(2'-hydroxyphenyl)benzimidazole(hpbm) is an N,*O* bidentate ligand that comprises two donor groups of relevance to the coordination of metal centers in biological systems, namely phenolate (tyrosine) and imidazole (histidine). In present paper, we report the synthesis and crystal structure of the dimethylformamide solvate of the Co^II^ complex with two deprotonated ligands, [Co(pbm)_2_].2(DMF).

The structure of the complex is shown in Fig. 1. The molecules of the cobalt complex are disposed about a twofold symmetry axis. The Co—O and Co—N bond lengths are 1.9135 (2) Å, and 1.9784 (2)Å respectively (Table 1). The cobalt atoms adopt a distorted four-coordinate environment with a dihedral angle of 75.4 (3)° between the two coordinating ligands (as defined by the Co—N—O planes). The O—Co—O and N—Co—N bond angles are 131.26 (2)° and 123.60 (2)°. In the title complex, the stronger hydrogen bonds are formed through oxygen atom of DMF with uncoordinated nitrogen atom of the ligand. The weaker hydrogen bonds are formed through uncoordinated oxygen atom of the ligand with carbon atom of another ligand.Through above the interaction, a novel two-dimensional network structure was formed where we happened to find the solvent molecules inside.

### Experimental

The ligand, 2-(2'-hydroxyphenyl)benzimidazole, was synthesized as follows: A solution of 2.32 g (19 mmol) of salicylaldehyde in 15 ml of EtOH was added to a solution of 2.05 g (19 mmol) of *o*-phenylenediamine in 25 ml of EtOH with stirring and heating. The resulting orange solution was refluxed for hour and then cooled to room temperature. After standing in the refridgerator for 12 h, the orange solution was filtered and 15 ml of ether was added to the solution. Standing in the open air for 2 d yielded orange crystalline needles which were filtered and air-dried. Yield: 60%. The elemental analysis results are completely in agreement with the structural composition of the ligand. m.p. 524–525 K.

The title complex was obtained as follows: To a filtered solution of HL(0.420 g, 2 mmol) and KOH (0.112 g, 2 mmol) in methanol(60 ml) at rt was added a filtered solution of Co(OAc)_2_^.^H_2_O (0.250 g, 1 mmol) in methanol(29 ml) with stirring. The product began to crystallize from the solution tardily. After 1 h the pink solid was filtered off, washed with methanol, and air-dried. X-ray quality single crystals were grown by the vapour diffusion of ether into a DMF solution of the solid above to yield pink crystals of the title complex. Analysis. Calcd for C_32_H_32_N_6_O_4_Co (%): C 61.64, H 5.14, N 13.48. Found (%): C 62.06, H 4.95, N 13.86.

### Refinement

The H atoms on C atom were treated as riding with C—H = 0.96Å and *U*_iso_(H) = 1.5U*_e.g_* of the parent atom. The H atoms on N atom were refined with *U*_iso_(H) = 1.2U*_e.g_* of the parent atom and N—H = 0.86 Å. The final electron density maximum and minimum were +0.31 and -0.46 e Å^-3^ respectively.

### Crystal data

**Table:**

[Co(C_13_H_9_N_2_O)_2_]·2C_3_H_7_NO	*D*_x_ = 1.391 Mg m^−^^3^
*M**_r_* = 623.57	Mo *K*α radiation, λ = 0.71073 Å
Orthorhombic, *P**b**c**n*	Cell parameters from 4622 reflections
*a* = 15.440 (2) Å	θ = 3.0–22.7°
*b* = 8.7022 (12) Å	µ = 0.62 mm^−^^1^
*c* = 22.156 (3) Å	*T* = 298 K
*V* = 2977.0 (7) Å^3^	Flake, pink
*Z* = 4	0.34 × 0.28 × 0.09 mm
*F*(000) = 1300	

### Data collection

**Table:**

Bruker SMART CCD area-detector diffractometer	3157 independent reflections
Radiation source: fine-focus sealed tube	2167 reflections with *I* > 2σ(*I*)
graphite	*R*_int_ = 0.036
ω scans	θ_max_ = 26.9°, θ_min_ = 2.3°
Absorption correction: multi-scan (ABSCOR; Higashi, 1995)	*h* = −18→18
*T*_min_ = 0.816, *T*_max_ = 0.946	*k* = −11→9
12837 measured reflections	*l* = −27→13

### Refinement

**Table:**

Refinement on *F*^2^	Primary atom site location: structure-invariant direct methods
Least-squares matrix: full	Secondary atom site location: difference Fourier map
*R*[*F*^2^ > 2σ(*F*^2^)] = 0.038	Hydrogen site location: inferred from neighbouring sites
*wR*(*F*^2^) = 0.107	H-atom parameters constrained
*S* = 1.03	*w* = 1/[σ^2^(*F*_o_^2^) + 0.8613*P*] where *P* = (*F*_o_^2^ + 2*F*_c_^2^)/3
3157 reflections	(Δ/σ)_max_ = 0.011
197 parameters	Δρ_max_ = 0.24 e Å^−^^3^
0 restraints	Δρ_min_ = −0.34 e Å^−^^3^

### Special details

**Table:**

Experimental. ABSCOR BY T.Higashi 8 march,1995
Geometry. All e.s.d.'s (except the e.s.d. in the dihedral angle between two l.s. planes) are estimated using the full covariance matrix. The cell e.s.d.'s are taken into account individually in the estimation of e.s.d.'s in distances, angles and torsion angles; correlations between e.s.d.'s in cell parameters are only used when they are defined by crystal symmetry. An approximate (isotropic) treatment of cell e.s.d.'s is used for estimating e.s.d.'s involving l.s. planes.
Refinement. Refinement of *F*^2^ against ALL reflections. The weighted *R*-factor *wR* and goodness of fit *S* are based on *F*^2^, conventional *R*-factors *R* are based on *F*, with *F* set to zero for negative *F*^2^. The threshold expression of *F*^2^ > σ(*F*^2^) is used only for calculating *R*-factors(gt) *etc*. and is not relevant to the choice of reflections for refinement. *R*-factors based on *F*^2^ are statistically about twice as large as those based on *F*, and *R*- factors based on ALL data will be even larger.

### Fractional atomic coordinates and isotropic or equivalent isotropic displacement parameters (Å^2^)

**Table:**

	*x*	*y*	*z*	*U*_iso_*/*U*_eq_	
Co1	0.5000	0.02008 (5)	0.2500	0.03984 (15)	
O1	0.40147 (10)	−0.07066 (19)	0.28840 (6)	0.0548 (4)	
O2	0.48308 (13)	0.3080 (2)	0.53185 (8)	0.0767 (6)	
N1	0.53688 (11)	0.12752 (19)	0.32438 (7)	0.0382 (4)	
N2	0.53681 (11)	0.2071 (2)	0.41924 (7)	0.0414 (4)	
H2A	0.5210	0.2194	0.4561	0.050*	
N3	0.51069 (13)	0.3913 (2)	0.62601 (8)	0.0522 (5)	
C1	0.60775 (12)	0.2234 (2)	0.33281 (9)	0.0371 (5)	
C2	0.67200 (14)	0.2690 (2)	0.29255 (10)	0.0473 (5)	
H2	0.6718	0.2360	0.2526	0.057*	
C3	0.73595 (15)	0.3649 (3)	0.31418 (11)	0.0548 (6)	
H3	0.7797	0.3977	0.2883	0.066*	
C4	0.73617 (16)	0.4136 (3)	0.37414 (11)	0.0588 (7)	
H4	0.7803	0.4781	0.3873	0.071*	
C5	0.67330 (16)	0.3694 (3)	0.41443 (10)	0.0542 (6)	
H5	0.6740	0.4019	0.4544	0.065*	
C6	0.60842 (13)	0.2734 (2)	0.39230 (9)	0.0396 (5)	
C7	0.49560 (13)	0.1191 (2)	0.37745 (8)	0.0343 (4)	
C8	0.41866 (13)	0.0275 (2)	0.39038 (9)	0.0374 (5)	
C9	0.38635 (14)	0.0222 (2)	0.44962 (10)	0.0449 (5)	
H9	0.4138	0.0795	0.4794	0.054*	
C10	0.31575 (15)	−0.0646 (3)	0.46486 (11)	0.0534 (6)	
H10	0.2960	−0.0666	0.5045	0.064*	
C11	0.27404 (15)	−0.1491 (3)	0.42092 (11)	0.0568 (6)	
H11	0.2257	−0.2076	0.4310	0.068*	
C12	0.30338 (15)	−0.1473 (3)	0.36265 (11)	0.0542 (6)	
H12	0.2741	−0.2045	0.3337	0.065*	
C13	0.37675 (13)	−0.0612 (3)	0.34506 (9)	0.0424 (5)	
C14	0.52757 (18)	0.3784 (3)	0.56829 (12)	0.0634 (7)	
H14	0.5772	0.4264	0.5539	0.076*	
C15	0.5674 (2)	0.4749 (3)	0.66682 (14)	0.0821 (9)	
H15A	0.5929	0.4045	0.6951	0.123*	
H15B	0.5344	0.5508	0.6883	0.123*	
H15C	0.6123	0.5245	0.6441	0.123*	
C16	0.43673 (18)	0.3165 (3)	0.65240 (12)	0.0732 (8)	
H16A	0.4056	0.2617	0.6217	0.110*	
H16B	0.3995	0.3921	0.6703	0.110*	
H16C	0.4557	0.2457	0.6829	0.110*	

### Atomic displacement parameters (Å^2^)

**Table:**

	*U*^11^	*U*^22^	*U*^33^	*U*^12^	*U*^13^	*U*^23^
Co1	0.0411 (3)	0.0512 (3)	0.0272 (2)	0.000	−0.00169 (17)	0.000
O1	0.0521 (10)	0.0778 (11)	0.0346 (8)	−0.0211 (8)	−0.0020 (7)	−0.0004 (8)
O2	0.0896 (14)	0.0933 (14)	0.0471 (11)	0.0015 (11)	0.0029 (10)	−0.0212 (10)
N1	0.0390 (10)	0.0456 (10)	0.0300 (9)	−0.0014 (8)	0.0011 (7)	0.0010 (7)
N2	0.0454 (10)	0.0508 (11)	0.0279 (9)	−0.0063 (8)	0.0026 (7)	−0.0028 (8)
N3	0.0615 (13)	0.0562 (12)	0.0388 (11)	0.0040 (10)	0.0039 (9)	−0.0020 (9)
C1	0.0357 (11)	0.0411 (11)	0.0346 (10)	0.0010 (9)	−0.0002 (9)	0.0021 (9)
C2	0.0478 (13)	0.0568 (14)	0.0374 (11)	−0.0011 (11)	0.0071 (10)	0.0027 (10)
C3	0.0463 (14)	0.0619 (15)	0.0561 (15)	−0.0103 (11)	0.0105 (11)	0.0023 (12)
C4	0.0506 (15)	0.0655 (16)	0.0604 (16)	−0.0200 (12)	−0.0003 (12)	−0.0033 (12)
C5	0.0577 (15)	0.0608 (15)	0.0440 (13)	−0.0130 (12)	−0.0006 (11)	−0.0083 (11)
C6	0.0390 (12)	0.0444 (12)	0.0355 (10)	−0.0025 (9)	0.0018 (9)	0.0016 (9)
C7	0.0379 (11)	0.0382 (11)	0.0269 (10)	0.0034 (9)	−0.0016 (9)	0.0019 (8)
C8	0.0351 (11)	0.0424 (12)	0.0348 (11)	0.0023 (9)	0.0000 (9)	0.0058 (9)
C9	0.0490 (14)	0.0492 (13)	0.0366 (12)	0.0030 (10)	0.0051 (10)	−0.0004 (10)
C10	0.0519 (14)	0.0624 (15)	0.0458 (13)	0.0038 (12)	0.0155 (11)	0.0097 (12)
C11	0.0433 (14)	0.0650 (16)	0.0620 (17)	−0.0087 (12)	0.0073 (12)	0.0149 (13)
C12	0.0443 (13)	0.0664 (16)	0.0521 (14)	−0.0113 (11)	−0.0065 (11)	0.0046 (12)
C13	0.0358 (12)	0.0517 (13)	0.0395 (12)	−0.0022 (10)	−0.0032 (9)	0.0091 (10)
C14	0.0687 (17)	0.0705 (18)	0.0510 (16)	0.0073 (14)	0.0124 (13)	0.0001 (14)
C15	0.094 (2)	0.083 (2)	0.0694 (19)	0.0018 (17)	−0.0113 (17)	−0.0150 (16)
C16	0.077 (2)	0.0775 (19)	0.0646 (17)	0.0041 (15)	0.0186 (15)	0.0129 (15)

### Geometric parameters (Å, °)

**Table:**

Co1—O1	1.9135 (15)	C4—H4	0.9300
Co1—O1^i^	1.9135 (15)	C5—C6	1.394 (3)
Co1—N1	1.9784 (16)	C5—H5	0.9300
Co1—N1^i^	1.9784 (16)	C7—C8	1.459 (3)
O1—C13	1.315 (2)	C8—C9	1.405 (3)
O2—C14	1.224 (3)	C8—C13	1.423 (3)
N1—C7	1.339 (2)	C9—C10	1.369 (3)
N1—C1	1.389 (2)	C9—H9	0.9300
N2—C7	1.360 (2)	C10—C11	1.379 (3)
N2—C6	1.383 (2)	C10—H10	0.9300
N2—H2A	0.8600	C11—C12	1.368 (3)
N3—C14	1.310 (3)	C11—H11	0.9300
N3—C16	1.439 (3)	C12—C13	1.413 (3)
N3—C15	1.454 (3)	C12—H12	0.9300
C1—C6	1.388 (3)	C14—H14	0.9300
C1—C2	1.392 (3)	C15—H15A	0.9600
C2—C3	1.379 (3)	C15—H15B	0.9600
C2—H2	0.9300	C15—H15C	0.9600
C3—C4	1.394 (3)	C16—H16A	0.9600
C3—H3	0.9300	C16—H16B	0.9600
C4—C5	1.374 (3)	C16—H16C	0.9600
			
O1—Co1—O1^i^	131.26 (11)	N1—C7—C8	126.16 (18)
O1—Co1—N1	93.07 (6)	N2—C7—C8	123.70 (17)
O1^i^—Co1—N1	109.66 (7)	C9—C8—C13	118.69 (19)
O1—Co1—N1^i^	109.66 (7)	C9—C8—C7	119.39 (19)
O1^i^—Co1—N1^i^	93.07 (6)	C13—C8—C7	121.88 (18)
N1—Co1—N1^i^	123.60 (10)	C10—C9—C8	122.1 (2)
C13—O1—Co1	129.02 (13)	C10—C9—H9	118.9
C7—N1—C1	106.85 (16)	C8—C9—H9	118.9
C7—N1—Co1	124.63 (14)	C9—C10—C11	119.5 (2)
C1—N1—Co1	128.51 (13)	C9—C10—H10	120.3
C7—N2—C6	108.38 (16)	C11—C10—H10	120.3
C7—N2—H2A	125.8	C12—C11—C10	120.3 (2)
C6—N2—H2A	125.8	C12—C11—H11	119.8
C14—N3—C16	121.1 (2)	C10—C11—H11	119.8
C14—N3—C15	122.0 (2)	C11—C12—C13	122.1 (2)
C16—N3—C15	116.8 (2)	C11—C12—H12	118.9
C6—C1—N1	108.77 (17)	C13—C12—H12	118.9
C6—C1—C2	120.93 (19)	O1—C13—C12	117.6 (2)
N1—C1—C2	130.30 (19)	O1—C13—C8	125.17 (19)
C3—C2—C1	117.4 (2)	C12—C13—C8	117.24 (19)
C3—C2—H2	121.3	O2—C14—N3	125.1 (3)
C1—C2—H2	121.3	O2—C14—H14	117.5
C2—C3—C4	121.1 (2)	N3—C14—H14	117.5
C2—C3—H3	119.4	N3—C15—H15A	109.5
C4—C3—H3	119.4	N3—C15—H15B	109.5
C5—C4—C3	122.2 (2)	H15A—C15—H15B	109.5
C5—C4—H4	118.9	N3—C15—H15C	109.5
C3—C4—H4	118.9	H15A—C15—H15C	109.5
C4—C5—C6	116.6 (2)	H15B—C15—H15C	109.5
C4—C5—H5	121.7	N3—C16—H16A	109.5
C6—C5—H5	121.7	N3—C16—H16B	109.5
N2—C6—C1	105.86 (17)	H16A—C16—H16B	109.5
N2—C6—C5	132.3 (2)	N3—C16—H16C	109.5
C1—C6—C5	121.82 (19)	H16A—C16—H16C	109.5
N1—C7—N2	110.13 (17)	H16B—C16—H16C	109.5
			
O1^i^—Co1—O1—C13	117.1 (2)	C1—N1—C7—N2	0.6 (2)
N1—Co1—O1—C13	−2.6 (2)	Co1—N1—C7—N2	−179.44 (13)
N1^i^—Co1—O1—C13	−130.02 (19)	C1—N1—C7—C8	−178.17 (18)
O1—Co1—N1—C7	0.38 (17)	Co1—N1—C7—C8	1.8 (3)
O1^i^—Co1—N1—C7	−135.71 (16)	C6—N2—C7—N1	−0.9 (2)
N1^i^—Co1—N1—C7	116.49 (17)	C6—N2—C7—C8	177.94 (18)
O1—Co1—N1—C1	−179.71 (17)	N1—C7—C8—C9	175.51 (19)
O1^i^—Co1—N1—C1	44.19 (18)	N2—C7—C8—C9	−3.1 (3)
N1^i^—Co1—N1—C1	−63.60 (15)	N1—C7—C8—C13	−2.3 (3)
C7—N1—C1—C6	−0.1 (2)	N2—C7—C8—C13	179.04 (19)
Co1—N1—C1—C6	179.93 (14)	C13—C8—C9—C10	−0.4 (3)
C7—N1—C1—C2	179.2 (2)	C7—C8—C9—C10	−178.3 (2)
Co1—N1—C1—C2	−0.7 (3)	C8—C9—C10—C11	−0.5 (3)
C6—C1—C2—C3	0.1 (3)	C9—C10—C11—C12	0.5 (4)
N1—C1—C2—C3	−179.1 (2)	C10—C11—C12—C13	0.4 (4)
C1—C2—C3—C4	0.2 (4)	Co1—O1—C13—C12	−176.47 (16)
C2—C3—C4—C5	−0.2 (4)	Co1—O1—C13—C8	2.8 (3)
C3—C4—C5—C6	−0.3 (4)	C11—C12—C13—O1	177.9 (2)
C7—N2—C6—C1	0.8 (2)	C11—C12—C13—C8	−1.4 (3)
C7—N2—C6—C5	−178.3 (2)	C9—C8—C13—O1	−177.9 (2)
N1—C1—C6—N2	−0.4 (2)	C7—C8—C13—O1	−0.1 (3)
C2—C1—C6—N2	−179.79 (19)	C9—C8—C13—C12	1.3 (3)
N1—C1—C6—C5	178.78 (19)	C7—C8—C13—C12	179.18 (19)
C2—C1—C6—C5	−0.6 (3)	C16—N3—C14—O2	2.0 (4)
C4—C5—C6—N2	179.6 (2)	C15—N3—C14—O2	179.0 (3)
C4—C5—C6—C1	0.7 (3)		

Symmetry codes: (i) −*x*+1, *y*, −*z*+1/2.

### Hydrogen-bond geometry (Å, °)

**Table:**

*D*—H···*A*	*D*—H	H···*A*	*D*···*A*	*D*—H···*A*
N2—H2A···O2	0.86	1.94	2.772 (2)	164.
